# Current Understanding of “Mixed Corticomedullary Adrenal Tumor” and an Insight into Genomic Profiling

**DOI:** 10.3390/clinpract12060096

**Published:** 2022-11-11

**Authors:** Asad Ullah, Farah Ayman Elsaid Mohamed, Jaffar Khan, Katharine Tracy, Muhabat Khan, Samiha Mohsen, Abdul Qahar Khan Yasinzai, Kaleemullah Badini, Philip T. Sobash, Saleh Heneidi, Nagla Abdel Karim

**Affiliations:** 1 Department of Pathology, Vanderbilt University Medical Center, Nashville, TN 37232, USA; 2 Department of Internal Medicine College of Medicine, Cairo University, Cairo 12613, Egypt; 3 Department of Pathology and Laboratory Medicine, Indiana University School of Medicine, Indianapolis, IN 46202, USA; 4 Department of Medicine, Medical College of Georgia, Augusta University, Augusta, GA 30912, USA; 5 Department of Medicine, Bolan Medical College, Sandman Provincial Hospital, Quetta 83700, Pakistan; 6 Department of Medicine, University of Calgary, Calgary, AB T2N 1N4, Canada; 7 Department of Pathology, Cedars Sinai Medical Center, Los Angeles, CA 90048, USA; 8 Department of Hematology/Oncology, Inova Schar Cancer Institute, University of Virginia, Fairfax, VA 22031, USA

**Keywords:** mixed corticomedullary adrenal tumor (MCMT), collision theory, adrenal tumor

## Abstract

Background: Malignant mixed corticomedullary adrenal tumors (MCMTs) are extremely rare, with limited cases reported in the literature. The pathophysiology of malignant MCMTs is not well understood; the most prevailing theories are that it is a composite tumor of embryologically derived mesodermal (adrenal cortex) and neural crest (medulla) origin, perpetuating as two distinct cell lines forming a singular mass. Clinical features and laboratory diagnosis are associated with hypersecretions of the adrenal cortex and medulla. Surgical resection is curative in an isolated tumor. We reviewed and compared cases in the literature highlighting the pathogenesis and genetics of benign and malignant MCMT. Methods: Comprehensive literature analysis was conducted on PubMed and all the cases of mixed corticomedullary adrenal tumor published in English were included. Results: Most patients were female (73.1%) with a median age of 49 in women and 50 in men. Surgery was performed in all patients, and in four patients with malignant disease, chemotherapy was used as well. Clinically, most patients presented with hypertension (69%) followed by Cushing syndrome (42%) and diabetes (19%). Tumors often produced cortisol (74%), catecholamines (50%), and adrenocorticotrophic hormone (ACTH) (38%), with lower incidence of aldosterone- (7%) or dopamine (4%)-producing tumors. Immunohistochemical staining of 96% of cases showed Chromogranin-A (73%) and Synaptophysin (62%), followed by Inhibin-α (50%), Melan-A (31%), and S-100 (23%). Of the reported four cases with malignant disease, three showed a Ki-67 index of 40–50% with one showing less than 5%. Conclusion: Mixed corticomedullary adrenal tumors rarely present as a malignant disease requiring chemotherapy. Most MCMTs confer a good prognosis and respond well to surgical resection, though their pathogenesis is largely up to speculation because of limited data. Current theories regarding MCMT pathogenesis should be investigated further with genetic testing. Future research on MCMT may provide ways to guide physician diagnosis and subsequent treatment for refractory cases.

## 1. Introduction

Mixed corticomedullary tumors (MCMT) were first described by Mathison and Waterhouse in 1969 [1]. They are described as such due to their histological distinctions of adrenal cortical and medullary components. While patterns emerge as to the symptoms, they are non-pathognomic and not known to be associated with other underlying etiologies. The presenting symptoms are in form of Cushing syndrome, hypertension, hyperaldosteronism, hypokalemia, paroxysmal hypertension, and hypertension emergency [1,2,3,4,5]. It is difficult to obtain a preoperative diagnosis due to the rare nature, and various presenting multisystem symptoms.

Treatment consists of resection the tumor through adrenalectomy. Surgical resection results in symptomatic relief. Few cases in the literature demonstrate any malignant potential associated with MCMTs, although it has been seen in more recent decades. The etiology and mechanisms(pathophysiology) of these tumors is not well understood. Many proposed mechanisms have been cited such as “collision theory”, overproduction of adrenocorticotrophic hormone (ACTH) in a pheochromocytoma, pluripotent stem cells, or an underlying oncogene component [6,7,8,9]. Given the scarcity at which MCMT is seen, it is difficult to study these tumors in a full investigative fashion.

## 2. Materials and Methods

The review was conducted through PubMed with the search terms “corticomedullary”, “mixed”, and “adrenal”. Inclusion criteria were based on reports evaluated on having reported a case of mixed corticomedullary tumor(s). There were no exclusion criteria.

## 3. Results

Mixed corticomedullary tumors have a predilection in female patients (19/26), with mixed results between sides that would be expected for bilateral organs. The age range for all cases (male and female) was from 25 to 78. The median age for women (n = 19) and men (n = 7) was 49 and 50 years. The initial presenting symptoms were variable, with hypertension in 18/26 (69%) and Cushing syndrome in 11/26 (42%) being the most common (Table 1). Diabetes was present in 5/26 (19%) patients, and one patient presented with gestational diabetes. Four cases presented with flank pain, ipsilateral in three and contralateral in one patient. Tumor size is variable, with the smallest measuring 2.5 × 2.4 × 2.0, and the largest 22 cm.

Hormone production varied slightly among tumors. The most common was cortisol production 73% (19/26) and catecholamines 50% (13/26). ACTH production was high in 38% (10/26), aldosterone in 7% (2/26), and dopamine in one case (4%). Immunohistochemical staining was performed in 25/26 cases (96%). Chromogranin-A and synaptophysin were the most commonly seen in 19/26 (73%) and 16/26 (62%) cases, respectively. Adrenal cortical staining was shown most often with Inhibin-α 13/26 (50%), Melan-A 8/26 (31%), S-100 6/26 (23%). Less common were calretinin and INMS1. In one case, CYP17, CYP11 β, TH, DBH, and PNMT were used (Table 2).

Malignant features were seen in 15% (4/26) of cases. Tumor necrosis was observed in these malignant cases. Three-fourths of cases showed a Ki-67 index, with one showing <5%, and the others 40–50%. Three studies had genetic sequencing performed to identify an underlying molecular alteration for these tumor presentations. Additionally included in this review were two studies where the tumors were seemingly “mixed” but were not described as the other tumors. Delevaux described it as a “dual tumor”, whereas Duan described the tumor as having a “distinct arrangement of cortical and medullary layers”.

**Table 2:** Documented cases of mixed corticomedullary tumors (benign and malignant). TH (tyrosine hydroxylase); DBH (dopamine beta hydroxylase); PNMT (phenylethanolamine-N-methyltransferase; CYP17 (17 α -hydroxyl/17,20-lyase); 3β -HSD (3β-hydroxy-Δ5-steroid dehydrogenase).

Reference	Age	Sex	Clinical Features	Size (cm)	Hormones	Immunohistochemistry (+)	Malignant	Genetic Analysis	Concurrent Pathological Findings	Treatment
Mathison and Waterhouse [1]	39	F	Cushing syndrome, hypertensive crisis	4 × 3 × 3	Cortisol, ACTH, catecholamines	N/A	N	N	None	Left adrenalectomy
Akai [7]	61	F	HTN	3 × 3.5	Cortisol, ACTH, catecholamines	N/A	N	N	None	Right adrenalectomy
Ohta [10]	32	M	Cushing syndrome, HTN	4.5	Cortisol, ACTH	N/A	N	N	None	
Michal and Havlicek [11]	56 & 32	F	56—Cushing syndrome, hypertension 32—Cushing’s HTN	(56)—6 × 7 × 8 (32)—9 ×7 × 5	Cortisol, ACTH, aldosterone	Synaptophysin, chromogranin, EMA, 113-1	N	N	(56)—spindle cell carcinoma	Adrenalectomy
Delevaux [2]	47	M	HTN, diabetes	9	Aldosterone	NSE	N	N	None	Left adrenalectomy
Weinke [6]	34 & 52	F	34—HTN, hair loss, amenorrhea 52—Cushing syndrome, flank pain	(34)—4.5 (52)—4.5 × 2.5	(34)—Cortisol (52)—N/A	Synaptophysin, chromogranin, S-100, Inhibin-α	N	N	None	Right adrenalectomy
Chu [12]	55	F	Cushing syndrome	2.5 × 2.4 × 2.0	Cortisol, ATCH	Inhibin-α, Melan A, calretinin, chromogranin, synaptophysin	N	N	Ipsilateral myelolipidoma	Left adrenalectomy
Lee [3]	25	F	Weight gain, bitemporal headache, postpartum diabetes	3.2	Cortisol, ACTH, catecholamines	Inhibin-α, Melan A, chromogranin	N	N	Contralateral pheochromocytoma and ganglioneuroma	Right adrenalectomy
Ma [4]	41	F	Cushing syndrome, amenorrhea, weight gain, HTN	4 × 4	Cortisol, ACTH (catecholamines not measured)	Synaptophysin, chromagranin, Melan A, Inhibin-α	N	N	None	Left adrenalectomy
Kimura [5]	54	F	HTN, diabetes	5.3 × 4.9	Cortisol, catecholamines	Chromogranin A, P450c21	N	N	None	Left adrenalectomy
Ajmi [13]	34	F	Hirsutism, weight gain, amenorrhea	6 × 4	Cortisol	Chromogranin A	N	N	None	Right adrenalectomy
Alexadraki [14]	66	F	Mild HTN, subclinical Cushing syndrome	4.2 × 3.7 × 3.4	Cortisol, ACTH, catecholamines	Synaptophysin, chromogranin	N	N	None	Left adrenalectomy
Singh [15]	48	F	Weight gain, edema	8 × 7 × 9	Catecholamines	Synaptophysin, chromogranin, S-100, Inhibin-α		N	None	Right adrenalectomy
Lau [16]	64	F	HTN	3.6	Catecholamines	Synaptophysin, Melan A, Inhibin-α	N	N	None	Right adrenalectomy
Michalopoulos [17]	63	F	Weight loss, abdominal pain	8 × 7.5 × 4.5	Cortisol, ACTH, catecholamine	Synaptophysin, chromogranin, NSE, Vimentin, S-100, calretinin, CKAE1/AE3	Y	N	Ki67 <5% Microvascular and capsular invasion	Right adrenalectomy Chemotherapy: carboplatin and etoposide
Kaneko [18]	63	M	HTN, diabetes	3.5 × 2.6 × 2.6	Dopamine	Synaptophysin, chromogranin	N	N	None	Left adrenalectomy, partial nephrectomy
Donatini [19]	53	M	Right flank pain	5.5 × 4 × 3.5	Catecholamines	Synaptophysin, Melan-A, Inhibin-α	N	N	None	Left adrenalectomy
Turk [20]	78	F	HTN, dizziness	10	Androgens, catecholamines	Synaptophysin, chromogranin, Melan-A, Inhibin-α	Y	N	Ki67, 40–50%, tumor necrosis, vascular invasion Stage III with Stage IV metastasis in liver at day 121 postop	Left adrenalectomy Chemotherapy: carboplatin and etoposide
Thinzar [21]	48	M	Fatigue, HTN, bruising	3.9	Cortisol, catecholamines	Synaptophysin, chromogranin, S1-100, Melan-A, Inhibin-α	N	N	Ki-67, <1%	Left adrenalectomy
Alsabek [22]	50	M	Left flank pain, anorexia, weakness, weight loss	22	Cortisol	Chromogranin, S-100, Inhibin-α, calretinin	Y	N	Tumor necrosis, focal but no capsular invasion	Left adrenalectomy
Duan [23]	58	M	Subclinical Cushing syndrome, HTN, diabetes	3 × 3.8	Cortisol, catecholamines	SF-1, chromogranin	N	N	Spindle positive cells	Right adrenalectomy
Kanzawa [24]	31	F	Gestational HTN	3.8 × 2.4	Cortisol, catecholamines	Synaptophysin, chromogranin, 3B-HSD, CYP17, CYP11β 1, TH, DBH, PNMT, Inhibin-α, INSM1	N	Y	Ki-67 <1%	Right adrenalectomy
Chiou [9]	32	F	Cushing syndrome, hypertension	8.8	Cortisol	Chromogranin, SOX2, CD44, OCT4	N	Y	None	Right adrenalectomy
Ramírez-Rentería [8]	16	F	Cushing syndrome, HTN	9.3 × 7.6 × 10.4	ACTH, cortisol	Inhibin A, Melin A, calretinin, ACTH, chromogranin A, synaptophysin, PS-100	Y	Y	Metastasis, Ki-67: 40%	Right adrenalectomy

+ (positive) N/A (not available), N (no), Y (yes), NSE (neuron specific enolase), () corresponds to age of the patient, & (and).

## 4. Discussion

Mixed corticomedullary tumors are a rare subset of adrenal tumors that display an appearance and clinical signs of adrenal cortical adenoma and pheochromocytoma. Seldomly they can be seen with other adrenal tumors, ipsilaterally and contralaterally. Most of the tumors reviewed were diagnosed postoperatively upon histological and immunohistochemical analysis. While there is a variation among the clinical presentations with these tumors, macroscopically the variation is not as broad. First termed “mixed corticomedullary tumors” by Weinke [6], due to their interspersed and mixed histological appearance in a single tumor, there are reports of a single tumor representing distinctly separate histological cortical and pheochromocytoma elements in the same encapsulated tumor in two cases [2,23]. We have included them in this review while not fitting the classic definition, as they were not macroscopically distinct and in a single encapsulation.

### Diagnosis and Associated Features

Preoperative diagnosis is limited in these tumors due to their rarity and symptoms mimicking adrenal cortical, pheochromocytoma, or neither. The most common symptoms at presentation were Cushing syndrome, hypertension, and diabetes, of which many resolved completely after adrenalectomy. Postoperative cortisol and catecholamine levels in many cases returned to normal limits. A majority of cases were described as incidentalomas, in which the patient may have had hypertension and diabetes. Other concurrent tumors were found in some cases such as myelolipoma, spindle cell carcinoma, and ganglioneuroma. It is unclear if there is any association, or if this is a coincidental finding. In the case reported by Lau [16], the patient was noted to have a history of neurofibromatosis and breast cancer. Interestingly, this is the only case to have described a previous diagnosis of cancer in someone with MCMT. Kanzawa et al. [24] speculated there may be a potential underlying oncogenic mutation that could be understood between the two cancers, but this is difficult to interpret with a few cases [25,26].

The radiological findings are nonspecific, such as heterogeneously enhancing lesions and overlap with findings of pheochromocytoma. However, ^123^I-meta-iodobenzylguanidine (MIBG) scintigraphy show increased focal uptake [16,24,27,28].

## 5. Conclusions

This is the largest review of all cases in the literature to date and the only one to compare benign and malignant MCMTs. It is also the first to report this specific tumor type in a child. The authors propose to group cases such as Dele Vaux and Duan as mixed corticomedullary tumors due to the nature of both histological subsets being demonstrated in a single encapsulated tumor. As the pathogenesis of the disease state is uncertain, it is unclear why these tumors developed in discreet histological entities rather than truly mixed as seen in others. As genetic sequencing becomes readily available, there may be more cases reported to help elucidate any underlying genomic target for these tumors, specifically those with malignant potential. To better understand any genetic component that may be targeted, we recommend casting a wide array and comparing it to other adrenal and renal tumors in adults and children for further clarity and additions to the literature. Most MCMTs are benign and have a good prognosis after surgical intervention. Only a few cases were described with malignant features and metastasis requiring chemotherapy. Further investigation should be performed when these seldom-seen tumors are discovered to elucidate further information on pathogenesis and possible treatment for those that may recur despite resection.

## Figures and Tables

**Table 1:** Characteristics of MCMT.

	Number of Cases	%
Sex		
Female	19	73.1
Male	7	26.9
Age		
Female	25–78	Median—49
Male	32–63	Median—50
Treatment		
Adrenalectomy/Nephrectomy	26	100
Chemotherapy (malignant cases)	4	100
Tumor Pathology		
Truly Mixed	24	92
Benign	22	87
Malignant	4	15
Metastasis	2	7.6
Concurrent Tumors	4	15
Genetic Analysis	4	15
Stem Cell Activity	2	8
